# Sealing Capability and SEM Observation of the Implant-Abutment Interface

**DOI:** 10.1155/2011/864183

**Published:** 2011-07-02

**Authors:** Fabio C. Lorenzoni, Paulo G. Coelho, Gerson Bonfante, Ricardo M. Carvalho, Nelson R. F. A. Silva, Marcelo Suzuki, Thelma Lopes Silva, Estevam A. Bonfante

**Affiliations:** ^1^Department of Prosthodontics, Integrated Center for Research, Bauru School of Dentistry, University of São Paulo, 17012-901, Bauru, SP, Brazil; ^2^Department of Biomaterials and Biomimetics, New York University College of Dentistry, New York, NY 10010, USA; ^3^Department of Prosthodontics, New York University College of Dentistry, New York, NY 10010, USA; ^4^Department of Prosthodontics, Tufts University School of Dental Medicine, Boston, MA 02111, USA; ^5^Department of Biological Science, Bauru School of Dentistry, University of São Paulo, 17012-901 Bauru, SP, Brazil

## Abstract

To evaluate the sealing capability of external hexagon implant systems and assess the marginal fit, two groups (*n* = 10 each) were employed: SIN (Sistema de Implantes Nacional, Brazil) and Osseotite, (Biomet 3i, USA). Sealing capability was determined by placing 0.7 *μ*L of 1% acid-red solution in the implant wells before the torque of their respective abutments. Specimens were then placed into 2.5 mL vials filled with 1.3 mL of distilled water with the implant-abutment interface submerged. Three samples of 100 *μ*L water were collected at previously determinate times. The absorbance was measured with a spectrophotometer, and the data were analyzed by Two-way ANOVA (*P* < .05) and Tukey's test. Marginal fit was determined using SEM. Leakage was observed for both groups at all times and was significantly higher at 144 hrs. SEM analysis depicted gaps in the implant-abutment interface of both groups. Gaps in the implant-abutment interface were observed along with leakage increased at the 144 hrs evaluation period.

## 1. Introduction

The use of dental implants has revolutionized prosthodontics and the fixed treatment options that can be offered to patients. High survival rates and long-term predictability for clinically loaded endosseous implants have been consistently reported resulting in one of the most successful treatment modalities in dentistry [1–4]. However, despite the early characterization of factors related to implant fixture success, it is noticeable that the understanding of prosthetic-related failures has been less explored [5]. Typically, an implant-supported rehabilitation is comprised by an endosseous implant that connects to a transmucosal abutment (2-piece), which receives the single or multiple unit prosthetic restoration. The location of this connection can be either submerged or at bone crest level or nonsubmerged. Regardless of location and type of connection (internal or external), it is important that the best implant-abutment interface fit is achieved in order to favor the stress distribution between connecting components and biological response, hindering microorganism colonization at this interface [6–8]. 

The most commonly used internal or external connections involve the use of a screw to clamp implant fixture and abutment. The stability of this connection is secured through a clamping force [9] which is challenged by unclamping forces derived from occlusal function. According to bolted joint mechanics, to achieve and maintain the stability of the screw-type connection, it is important that the gap size is minimum, which will decrease the likelihood of screw loosening [9]. It has been demonstrated that when gaps were minimized, the chances of screw loosening also decreased [9–11], thus showing the positive relationship between gap size and screw loosening.

Clinically, the absence of a gap-free implant-abutment interface can induce biological and mechanical complications, jeopardizing the implant long-term success. An intense host immunological response (acute inflammatory process) has been found at or near to gaps around implant-abutment interface [12], leading to a potential bone loss [12–14]. The bacterially contaminated interface may elicit and maintain an inflammatory process (peri-implantitis) [15, 16]. Such bacterial colonization may initiate during the surgical placement of the implant, the reopening, and installation of an intermediary or through the misfit of the prosthetic connection [7, 12, 13, 17]. Thus, as the interface gap allows fluid and bacterial microleakage [6, 18–20], the implant well may serve as a bacterial reservoir that allows microorganisms to seep in and out, perpetuating a peri-implantitis disease [7]. However, implant design junctions have changed over time, providing a more predictability of bone stability as displayed by one-piece implant [17] and by switching platform concept [21–25]. These positive reports may intensify the clinical negative impact provided by marginal misfit, when the implant-abutment interface is located at or below the alveolar crest [26]. 

From a mechanical perspective it has been identified in a systematic review that the most common technical complication of a single-unit implant-supported reconstruction is abutment or occlusal screw loosening, with a cumulative incidence of 12.7% after 5 years of followup [27]. One potential reason for this type of failure could be an ill-fitted implant-abutment interface destabilizing the implant-abutment connection [28–32]. 

Despite the outstanding success rates of modern implantology, its progress is sustained and driven towards the decrease of reported clinical failures. Several factors are speculated to play a role in the implant restorations longevity. One crucial factor is the maintenance of bone level on the long term, which can be negatively affected by mechanical and biological complications. Since the establishment of peri-implant bone level after the healing period is somewhat predictable, its stability over time can be affected by inherent issues in the implant-abutment connection [33, 34] from a mechanical [9–11, 29, 31, 32] and biological perspective [12, 17, 35–40]. 

Several methods have been proposed to assess the implant-abutment interface concerns. Studies have most commonly investigated the sealing capability to bacterial [7, 8, 14, 41] or color marker migration towards or from the implant well [6]. Direct observations of the implant-abutment interface have also been performed by X-ray [42], scanning electron microscopy (SEM) [43, 44], and optical microscopy [45]. Another possibility is the cross-sectional analysis and evaluation of the misfit made as a function of implant radius, which allows a more comprehensive observation of the adaptation along the implant-abutment interface [46]. However, the evaluation of implant-abutment sealing capability followed by SEM direct observation has not been addressed to date. 

This study sought to compare the sealing capability and marginal fit of two external hexagon implant systems by spectrophotometric quantification of microleakage at several incubation times followed by SEM observation of the implant-abutment interface.

## 2. Material and Methods

Two external hexagon implant systems (4.1  mm diameter) were used for this study (*n* = 10 per system: TryOn-SIN, Sistema de Implantes Nacional, São Paulo, SP, Brazil and Osseotite-Biomet 3i, Palm Beach, Fla, USA). The implants and their proprietary abutments were first subjected to the sealing capability testing and then to direct SEM observation of the interface.

### 2.1. Sealing Capability

**Figure 1:**
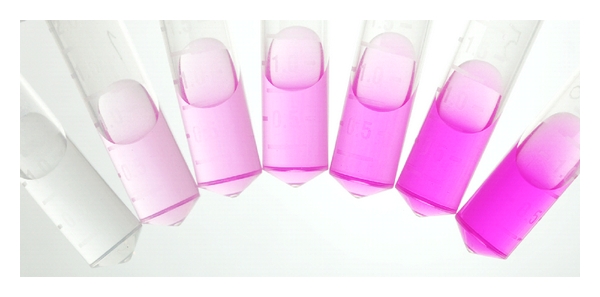
Increments to 0.1 *μ*L (more left) to 0.7 *μ*L (more right) added in 1.3 mL of the distillated water were used to compose the absorbance curve. The start point for this curve was the absorbance value of the water without addition of the color marker.

In order to quantify the amount of the color marker (1% acid-red in propylene glycol hydrosoluble pigment) (Caries Detector, Kuraray Medical Incorporation, Okayama, Japan) dissolved in the distilled water, a calibration curve was determined through linear regression (best line fit) using a fraction of a color marker volume in water. Seven color marker increments of 0.1 *μ*L (to 0.7 *μ*L) were added using an automated pipette (Eppendorf Research Pro, Westbury, USA) to 1.3 mL of distilled water placed in 2.5 mL vials. The absorbance of these color marker increments dissolved in water (Figure 1) was quantified with a spectrophotometer calibrated to a wavelength of 560 nm (Fluostar Optima—BMG, Labotech, Offenburg, Germany). The maximum amount of 0.7 *μ*L was determined from a pilot study which indicated that this volume was enough to fill the implant well and remained free of contact with the torqued abutment screw most apical portion. Samples from each increment (*n* = 5 per increment) were analyzed in the spectrophotometer calibrated to a wavelength of 560 nm to acquire the absorbance values, which were used to compose the absorbance curve. The starting point to formulate the absorbance curve was pure distilled water without color marker.

In the most apical portion of the implant well 0.7 *μ*L amount of color marker was dispensed by means of an automated pipette. Subsequently, the implants were held by a vise connected to a bench in a vertical position where the abutments were assembled onto the implants and torqued to 20 Ncm (as per manufacturers recommendations) using a hand torque wrench (TMEC, SIN—Sistema de Implantes, São Paulo, Brazil). The connected implants were placed into 2.5 mL vials (Eppendorf Research Pro, Westbury, USA) filled with 1.3 mL of distilled water assuring that the implant-abutment interface remained immersed, but not the interface between abutment and screw. The capped vials containing the implants and water were then kept at room temperature.

Using an automated pipette, samples of 100 *μ*L (*n* = 3 for each implant) were acquired at 1, 3, 6, 24, 48, 72, 96, and 144 hrs incubation time at room temperature. Each sample was transferred from the respective vial to a microplate (Costar 96, Costar—Switzerland) for absorbance evaluation. Immediately after that, the contents of the microplate were returned to the vials containing the implants. The arithmetic average of the three absorbance values was determined and used for statistical analyses. Two-way ANOVA at 95% level of significance and Tukey's test for multiple comparisons were utilized.

### 2.2. SEM Evaluation

Specimens were subjected to marginal fit evaluation in the SEM (Model 3500S, Hitachi Ltd., Osaka, Japan) at a 15 Kv acceleration voltage and 750x magnification. The inspection involved the search for marginal gaps.

## 3. Results

**Figure 2:**
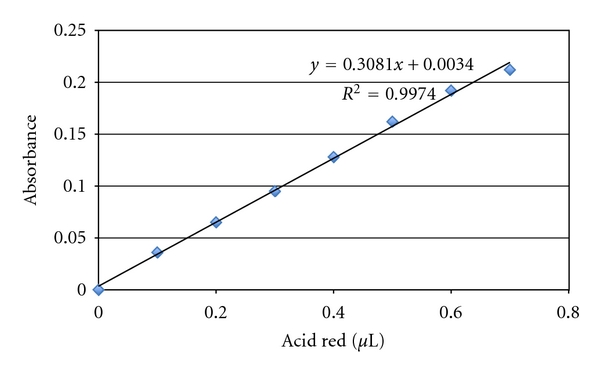
The absorbance curve was used to quantify the amount of the color marker release through the connection between implant-abutment.

The calibration curve generated by the 0.1 *μ*L increments of the color marker (up to 0.7 *μ*L) dissolved in water was linear presenting a *R* ^2^ of 0.9974 (Figure 2). 

**Figure 3:**
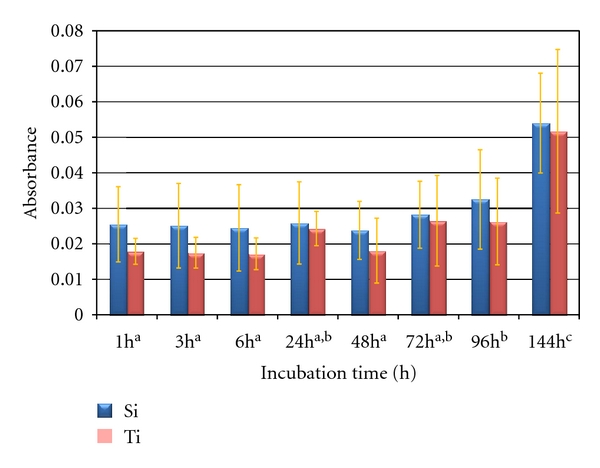
Color marker release as a function of incubation time.

The color marker release quantification showed no statistical difference (*P* > .05) between groups. However, both groups increased the amount of color marker release as a function of incubation time (Figure 3). No significant difference was observed between 1 up to 96  hours incubation times. The highest amount of color marker release was observed at 144  hours relative to all previous incubation times (*P* < .000001) for both groups. 

**Figure 4:**
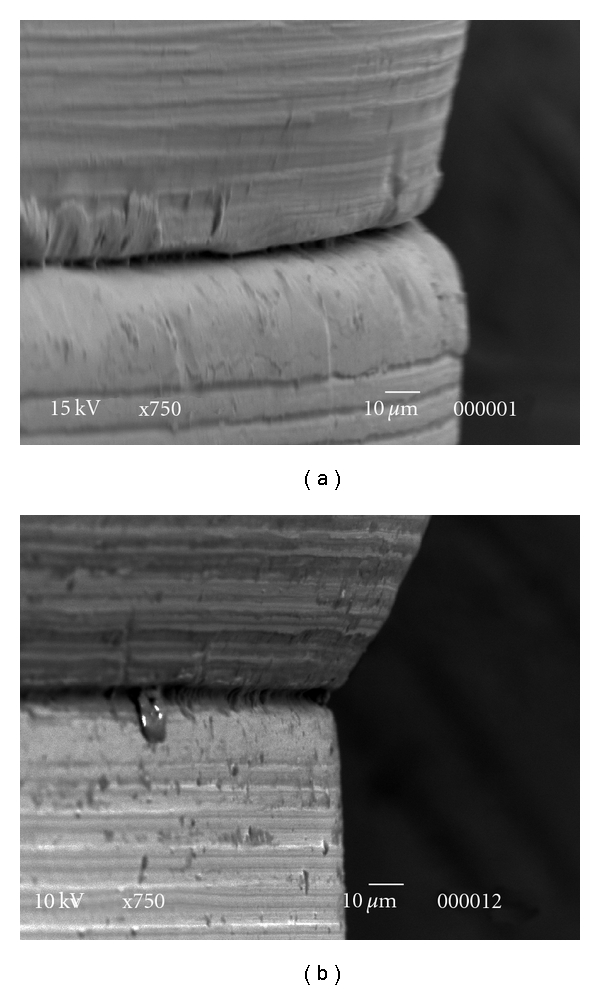
SEM Pictures of the marginal fit of interface implant-abutment of a representative specimen of SIN (a) and 3i (b) after the sealing capability test.

Representative SEM micrographs of the implant-abutment interface are presented in Figure 4. Gaps were observed in both groups and around the same implant-abutment system.

## 4. Discussion

Although our results showed the presence of gaps during the SEM marginal observation of the interface, caution must be taken when only this technique is considered as a method to evaluate the fit of the joint, since variations in gap sizes have been shown to occur along the implant radius in cross-sectional observations of this interface [46]. In addition, whereas knowledge of the interface size can allow the understanding of the potential of the bacterial colonization, it is limited in providing more insight into the possibility of fluid passage through the implant-abutment interface.

When compared to the results of sealing capability testing of internal connecting systems, the present data shows leakage also occurring in the investigated external connection systems. The external hexagon connection was chosen for its long history of use and the plethora of data concerning its application [30, 47]. Although efforts to reduce leakage by the use of polymeric components in the interface have hindered but not eliminated bacterial colonization [48], only a screw less interference-fit implant-abutment connection has shown to restrain bacterial passage along its interface [7]. 

Besides the alteration in connection designs, positive outcomes in bone level maintenance, when compared with matching implant-abutment dimensions, have been noticed in clinical prospective studies by two distinct approaches. The first involves the positioning of the implant-abutment interface inward and away from the outer edge of the implant (platform switching concept) [21, 22, 24, 25, 49] and the second comprises the absence of the connection by the use of one-piece implants [50]. The results for the first may be mainly attributed to distance increase between abutment interface and bone level, perhaps decreasing the bone response and consequently the bone loss [23]. For one-piece implants, it is possibly related to the absence of interface gap. However, the application of the platform switching concept seems to be limited to larger-diameter implants (5.0 or 6.0 mm) of the prosthetic platform diameter. 

Considering that a relationship between extension of bone loss and the magnitude of the inflammatory process has been suggested [12], likely associated with the presence of microorganism in the implant-abutment interface [15, 16], there seems to be a direct relationship between peri-implant disease and interface gap [7, 15]. Therefore, alterations in connection designs have always gained attention in implant-supported prosthodontics where considerable effort has been devoted to improve the stability and minimize the implant-abutment interface gap [7, 35, 39, 46, 51].

Imperfections related to machining of implants components, excessive torque during abutment placement (which may allow the distortion of its parts), and in addition the misfit between implant-abutment are factors that have been related to interface gap origin [13]. Therefore, new studies involving the sealing capability of different connection systems combined with fatigue testing to evaluate the effect of misfit on systems mechanical performance may bring insight for the development of new connection designs.

## 5. Conclusions

Evaluation of the sealing capability of two different systems showed the passage of fluids in both groups, and both groups presented implant-abutment gaps in the SEM micrographs.
